# Stress beyond coping? A Rasch analysis of the Perceived Stress Scale (PSS-14) in an Aboriginal population

**DOI:** 10.1371/journal.pone.0216333

**Published:** 2019-05-03

**Authors:** Pedro Henrique Ribeiro Santiago, Rachel Roberts, Lisa Gaye Smithers, Lisa Jamieson

**Affiliations:** 1 Australian Research Centre for Population Oral Health (ARCPOH), Adelaide Dental School, The University of Adelaide, Adelaide, South Australia, Australia; 2 School of Psychology, The University of Adelaide, Adelaide, South Australia, Australia; 3 School of Public Health, The University of Adelaide, Adelaide, South Australia, Australia; University of Copenhagen, DENMARK

## Abstract

The history of colonization contributed to Aboriginal and Torres Strait Islanders becoming one of the most disadvantaged groups in Australia. The multiple social inequalities, and therefore the constant insecurities for many about low income, poor living conditions, unemployment, and discrimination, generate chronic stress in this population. In the Baby Teeth Talk Study, an oral-health randomized controlled trial, the Perceived Stress Scale (PSS-14) was administered to 367 pregnant Aboriginal women at baseline. The aim of the present study was to evaluate the validity and reliability of the PSS-14 in an Aboriginal population. The study analysed: (a) model fit; (b) dimensionality; (c) local dependence; (d) differential item functioning; (e) threshold ordering and item fit; (f) targeting; (g) reliability; and (h) criterion validity. The dimensionality analysis indicated a two-factor structure, with negatively and positively worded items clustering together and 21.7% (95% Agresti-Coull C.I. [17.8%, 26.2%]) statistically significant t-tests between the persons’ estimates. After the creation of composite items, the revised Perceived Distress (χ^2^ (21) = 11.74, p = 0.946) and Perceived Coping (χ^2^ (28) = 17.63, p = 0.935) subscales fitted the Rasch model. Reliability was modest (PersonSeparationIndex_distress_ = 0.72; PersonSeparationIndex_coping_ = 0.76). The latent correlation between the Perceived Distress and Perceived Coping subscales was r = 0.14. It is hypothesized that the social inequalities experienced by the Aboriginal population are so pronounced that even Aboriginal pregnant women that perceived themselves as coping well with life challenges ended up endorsing items regarding high levels of stress. The present research showed that a revised PSS-14 is a culturally valid and modestly reliable psychological instrument to measure stress in a population of pregnant Aboriginal women in Australia.

## Introduction

A history of colonization and genocide contributed to Aboriginal and Torres Strait Islanders becoming one of the most disadvantaged groups in Australia [1]. The multiple social inequalities, and therefore the constant insecurities for many about low income, poor living conditions, unemployment, and discrimination, generate chronic stress and impact on both physical and mental health [2]. The risk of being exposed to stressful life events is two to five times greater for Aboriginal Australians [3]. For example, one in five Aboriginal youth reported living in a family confronted with at least seven major stressful life events in the last year, such as death, arrest, incarceration, alcohol abuse, among others [4, 5]. When the stress is experienced by Aboriginal persons in childhood, it has been associated with an increase in the odds of depression, anxiety, dementia, and Alzheimer disease during the life-course [6].

One of the most affected groups is pregnant Aboriginal women [7], who have a two to three-fold increase in the odds of high psychological distress associated with experiencing stressful episodes (e.g. family violence) during pregnancy [8]. In a recent study, the prevalence of Aboriginal women that experienced stressful life events while pregnant ranged from 36% to 58%. For example, 49% of the Aboriginal women reported witnessing the death of at least one family member or close friend during their gestation [9]. Rates of teenage pregnancy were also higher among Aboriginal woman, with the low socioeconomic position of many teenage Aboriginal mothers making them particularly vulnerable to stressful situations [10]. Prenatal stress has been associated with negative child outcomes such as low birth weight and reduced length of pregnancy [11], alongside with increased risk of impaired cognitive development [12]. In-utero exposure to the stress hormone cortisol has also been associated with lower adult educational attainment and verbal cognition [13].

The stress experienced by Aboriginal Australians needs to be contextualized in relation to the long-term effects of colonization on their sense of personal control and the social support of their communities. The theoretical associations between stress, sense of personal control and social support have been extensively investigated in the general population. For example, when individuals have a high sense of personal control (i.e. the belief about being able to influence outcomes in life) and encounter stressful events, they are more prone to engage in active efforts to reduce the demands, instead of more passive or avoidant emotion-focused coping strategies [14]. Furthermore, when a person is faced with multiple problems that might exceed her/his individual coping capability, having social support leads to a perception that others will provide the necessary resources and attenuates the stress reaction [15]. These associations are especially relevant for understanding the stress experienced by Aboriginal Australians in the contemporary world. The intended dismantling of Aboriginal culture and the undermining of self-determination with respect to political and social decisions led individuals to lose the feeling of personal control over their lives. Previous research among Aboriginals living in Arnhem land, an isolated Aboriginal reserve in the northeast of Australia, has shown that perceived stress was negatively associated with mastery (i.e. the ability to influence outcomes, one important aspect of personal control) [1]. Additionally, the forced separations caused by decades of assimilation policies have undermined their societal cohesion. For Aboriginal Australians, individual emotional well-being cannot be disentangled from their connection with the well-being of the whole community. The social support derived from a complex kinship system reinforces Aboriginal persons’ cultural identity and protects against discrimination and negative interactions with non-Indigenous persons [16, 17].

Notwithstanding the high exposure to stressful life events, the investigation of its impact on Aboriginal and Torres Strait Islander well-being has been faced with conceptual and methodological challenges [18]. One fundamental concern is the development of psychological instruments that are valid for this group [19]. This concern was initially raised by Aboriginal leaders, who brought awareness to the fact that their experiences of happiness, suffering, and well-being should not be assumed as equivalent to the western conceptualization of “mental health” and needed to be understood through a culturally-sensitive framework [16, 19].

The usual practice in research has been the application of mainstream Western psychological instruments to Aboriginal Australians to investigate associations between the test results and health outcomes without a comprehensive evaluation of test psychometric functioning [19]. The assumption is that the psychological instrument applied in the Aboriginal population will retain its psychometric properties and be equally valid; however, if the construct being measured has meaningful differences in the Aboriginal culture, the interpretation of item responses is subject to construct bias [20, 21].

### The Perceived Stress Scale (PSS)

One psychological instrument that has been applied to measure stress in Indigenous populations is the Perceived Stress Scale (PSS). The PSS is the most widely used instrument to measure perceived stress [22], comprising 14 items in its original version (PSS-14) [23] and was developed based on the theoretical perspective of Lazarus [24]. The notion of perceived stress, which differs from earlier views that focused on the biological aspects of stress (e.g. secretion of corticoids and other hormones as a reaction to stressors) [25], postulates that stressor effects occur only when a situation is both *appraised as threatening* and there is a *perception of insufficient coping resources* [24, 26]. The PSS has been previously administered to Indigenous groups in Canada [27, 28] and the United States [29, 30]. However, in all cases, the PSS was applied without an evaluation of construct validity for the Indigenous culture and in one study it was simply assumed due to being “extensively validated” elsewhere [29].

With respect to non-Indigenous populations, the construct validity and psychometric properties of the PSS have been well-established (e.g.: USA, Japan, Mexico, France, Brazil, China, Qatar, among others) [31]. The cumulative evidence from psychometric studies indicates that the PSS (PSS-14 and PSS-10) has a two-factor structure composed of the positively-worded and negatively-worded items [22]. The two-factor dimensionality is consistent with Lazarus’s [24] theory of stress as appraisal and coping [32], and has been interpreted as “Perceived Distress” and “Perceived Coping” [33], although other terminologies such as “Perceived Helplessness” and “Perceived Self-efficacy” have been used [22]. Notwithstanding that Cohen [34], in the first factor analysis of the PSS-14, stated that for “purposes of measuring perceptions of stress, the distinction between the two factors was considered irrelevant”, the majority of subsequent empirical studies found evidence of two distinct psychological factors [22]. For example, while both factors predicted depression in women, only the “Perceived Helplessness” factor had predicted depression in men [33]. The PSS scores have been consistently correlated with depression and anxiety [31], and recent studies added to the evidence that the magnitude of these associations differs according to each dimension [35, 36].

Despite the large body of evidence, the two-factor dimensionality of the PSS is not a consensus among researchers [22] and has been challenged in recent studies [32, 37, 38]. In relation to the PSS-14, for example, the main concern is that the two-factor solution consistently accounted for less than 50% of the total variance [31]. Two studies found that a bi-factor structure with a specific factor for the “Perceived Coping” items better explained the items’ covariance than the two-factor structure [32, 37]. In addition, Medvedev, Krägeloh [38] showed that, after resolving local dependence by creating composite items, the PSS-10 exhibited a unidimensional structure.

Regarding internal consistency, the Cronbach’s alpha of the PSS-14 was >.70 in 11 of 12 examined studies and test-retest reliability was >.70 in 2 of 3 studies[31]. Six studies have also investigated Differential Item Functioning (DIF). The evidence of DIF related to gender is mixed and some studies indicated no gender-related DIF [22, 39]. Cole [40] reported that the PSS-10 items 3, 6, 7, 8 and 10 had DIF by gender and this result was partially confirmed by Gitchel, Roessler [41] who found DIF for the same items 3 and 4, in addition to items 1 and 6. Regarding education, evidence of DIF was found for PSS-10 items 3, 4, 8 and 9 [40], and the PSS-10 items 3 and 4 showed evidence of DIF by age [39]. Other sources of DIF investigated by previous studies were ethnicity and literacy [40, 42].

### The present research

In South Australia, the Perceived Stress Scale (PSS-14) was completed by 364 pregnant Aboriginal women in the Baby Teeth Talk Study (BTT). The Baby Teeth Talk Study, whose name was given by the study’s Aboriginal Reference Group, is a randomized controlled trial that aimed to investigate if implementing a culturally-sensitive intervention would reduce early childhood caries of Aboriginal children [43]. The study received approval from the University of Adelaide Human Research Ethics Committee, the Aboriginal Health Council of South Australia, the Government of South Australia and the Human Research Ethics Committees of three participating South Australian hospitals.

Considering the disproportionate rates of stressful life events experienced by Aboriginal Australians [3] and Aboriginal women [7, 8], it is necessary to develop or adapt psychological instruments that measure stress and are culturally valid for this group. In the context of the BTT randomized controlled trial, the results of the PSS-14 were not used to modify the intervention and had no direct consequences for the Aboriginal women (i.e. it was not a high-stakes scenario). The intention was to use the PSS-14 results to measure stress on a group rather than individual level and inform the effects of stress (i.e. the exposure) on future outcomes (e.g. children caries at age 2, nutrition, among others). Nonetheless, the aim of validating the PSS-14 is that future applications can measure stress in Aboriginal Australians in a variety of different contexts. To the best of our knowledge, there are no studies that have evaluated the psychometric properties of the PSS in any Indigenous culture, including an Aboriginal and Torres Strait Islander population. The aim of the present study is to evaluate if the PSS-14 is a valid and reliable measure of perceived stress in an Aboriginal population, using the Rasch Measurement Model [44]. The hypothesis that the PSS-14 is a valid and reliable measure will be evaluated through the analysis of the: (a) model fit; (b) dimensionality; (c) local dependence; (d) differential item functioning; (e) threshold ordering and item fit; (f) targeting; (g) reliability; and (h) criterion validity. The criterion validity will be evaluated by inspecting convergent and divergent validity of the PSS-14 with respect to the complementary measures of Sense of Personal Control Scale (SPCS) and the Social Support Scale (SSS) and concurrent validity with health behaviors. These complementary measures were chosen due to the role of these two theoretical constructs (i.e. sense of personal control and social support) in the stress experienced by Aboriginal Australians in the contemporary world.

## Methods

### Participants and procedures

The sample comprised of 367 pregnant Aboriginal women living in South Australia. Participants were recruited through referrals from a variety of sources including community services, Indigenous groups, and hospitals. Potential participants were provided with information about the study from health services providers and the study staff. For those interested in participating, the researchers explained the project in detail and answered questions. All participants were informed that participation was voluntary and that they could refuse or withdrawn to participate at any stage of the study without justification. The individuals who decided to participate were then asked to complete and sign a form expressing consent [45]. All participant provided signed informed consent.

The mothers’ average age was 24.9 years (SD = 5.9, range = 14–43). Regarding education, approximately 3% of mothers had not attended school or had only attended primary school, 70% of mothers had completed secondary school, 20% had completed Technical and Further Education (TAFE) or Trade qualifications, and 7% had completed university or post-graduate degrees. TAFE is the biggest provider of post-secondary education in Australia. Unlike universities, which are composed mostly of full-time students, TAFE institutions allow students to combine study and work, and encourage programs of apprenticeships and traineeships [46]. In respect to socio-economic position, approximately 53% of mothers were on the lowest quintile of the Index of Relative Socio-Economic Advantage and Disadvantage [47] and only 6% were on the top two quintiles, indicating a population that was largely socio-economically disadvantaged. Among the mothers, approximately 51% were current tobacco smokers, 27% former smokers and 22% had never smoke. Additionally, 10% reported currently drinking alcohol, 82% said they used to drink alcohol and 8% have never drunk alcohol. The psychological instruments were administered as part of a broader questionnaire to the Aboriginal mothers by four research staff (three Indigenous and one non-Indigenous) at baseline, and the data was later entered into password-protected databases.

### Measures

#### Initial pilot testing

As a recommended procedure in cultural adaptation [48], the PSS-14, SPCS and SSS were initially discussed and administered to a 15-member Aboriginal Reference Group, comprising Aboriginal community members and Aboriginal Infant Care workers. The reference group evaluated the items’ wording to ensure content and face validity with respect to an Aboriginal culture and suggested minor modifications. The modifications made, and the subsequent new wording of all PSS-14 items can be found in Table 1.

**Table 1:** New wording of the PSS-14 items after adaptation for an Aboriginal culture.

Item number	Item content*
1	… felt upset because of something that happened?
2	… felt like you couldn’t control the important things in your life?
3	… felt nervous or stressed?
4	…dealt well with life hassles?
5	… coped well with important changes in your life?
6	… felt able to handle your personal problems?
7	… felt things were going your way?
8	… felt unable to cope with all the things that you had to do?
9	… felt able to control irritations in your life?
10	… felt you were on top of things?
11	… felt angered because of things that happened outside of your control?
12	… found yourself thinking about all the things that you have to do?
13	… felt able to control how you spend your time?
14	… felt troubles were piling up so high that you could not deal with them?

Note.

*Every item started with the sentence “How often during the LAST YEAR have you….”

Subsequent to the pilot testing, the measures were then applied at the study baseline.

#### Primary measure

Perceived Stress Scale (PSS): PSS-14 items were responded to on a five-point Likert scale (0 = Not at all, 1 = Rarely, 2 = Sometimes, 3 = Fairly often, 4 = Very often). The PSS-14 was responded by 364 out of the 367 pregnant women. The positively worded items were reverse-scored prior to the analysis.

#### Complementary measures

The Sense of Personal Control Scale (SPCS): The Sense of Personal Control Scale is a 12-item scale developed by Lachman [49] to measure an individual’s sense of personal control. The original factorial analysis identified the two dimensions of Mastery (MS), individual’s beliefs regarding the ability to influence outcomes, and Perceived Constraints (PC), how outcomes are believed to be determined by external factors [49]. The items were responded to on a five-point rating scale (1 = Not at all, 2 = Rarely, 3 = Sometimes, 4 = Fairly often, 5 = Very often). The SPCS has been validated for an Aboriginal population [50].

The Social Support Scale (SSS): The Social Support Scale is composed of 4 items, each one designed to evaluate the emotional, appraisal, instrumental and informational domains of social support as theorized by House [51]. The items were responded to on a five-point rating scale (1 = Not at all, 2 = Rarely, 3 = Sometimes, 4 = Fairly often, 5 = Very often). The SSS has also been previously validated for an Aboriginal population [50].

Alcohol drinking and smoking status: Alcohol drinking status was measured with a single question “Alcohol drinking status” with three response categories (1 = Currently drink alcohol, 2 = Used to drink alcohol, 3 = Have never drunk alcohol). Similarly, smoking status was measured with the question “Smoking status” (1 = Currently smoke, 2 = Used to smoke, 3 = Never smoked). In the analysis of criterion validity, the categories of “Used to…” and “Never…” were collapsed since they represented the participants who weren’t currently smoking or drinking.

### Statistical methods

#### The Rasch measurement model

The Rasch Model is part of the family of Item Response Theory (IRT) psychometric models [52]. The Rasch model for ordered response categories [53] is a generalization of the original model for dichotomous items [44] and is displayed in Eq 1.

$$P\left(X_{vi}=x|\Theta =\theta \right)=\frac{e^{\theta_{v}x-\sum_{z=1}^{x}\beta_{iz}}}{\sum_{h=0}^{mi}e^{\theta_{v}h-\sum_{z=1}^{h}\beta_{iz}}}$$ (1)

Eq 1 indicates that the probability of the person *v* endorsing category *x* (e.g. “Strongly Agree”) of item *i* with a total of *m* categories is a function of the person’s latent trait θ and the item thresholds β_iz_. The *item thresholds* parameters β_iz_ are conceived in terms of the latent trait θ and represent in the latent trait scale the point of equal probability of choosing between two adjacent categories (e.g. “Agree” and “Strongly Agree”). Therefore, in the polytomous Rasch Model, the probability of endorsing the category of an item is a function of characteristics of the person (the latent trait θ) and characteristics of the item (the item thresholds β_iz_) [54].

The development of the Rasch Model aimed to establish in social sciences the fundamental properties of measurement found in the natural sciences [55]. One of the fundamental properties of measurement is invariance. In natural sciences, the measurement of processes that can be observed (e.g. height) or cannot be directly observed (e.g. temperature) is independent of the measurement instrument (e.g. ruler/thermometer) being used [56]. The Rasch Model is a psychometric model that has an analogous property (named objectivity): the comparison of two individuals regarding a latent trait, such as the *difference of the amount* of stress experienced by two persons, is independent of the psychological instrument being used [57]. The objectivity is derived from a unique characteristic of the Rasch Model in comparison to other IRT models, that it has a statistically sufficient total score [58]. A statistic S(X) (e.g. the total score) of a vector of observable variables X (e.g. the item responses) is considered sufficient for a parameter θ (e.g. the latent trait) if X and θ are conditionally independent given S(X) [59]. This means that the total score, which is the summation of the item responses, encompasses all the available information for statistical inference regarding the respondent’s latent trait; and, for example, no information would be added by knowing the respondent’s characteristics (e.g. male or female, smoker or non-smoker) [60]. Since the parameters of the Rasch model are mathematically symmetrical [61], the functioning of the items (i.e. their difficulty and hierarchical order) is also independent of the population [57].

The invariance of persons’ and items’ parameters is an *intrinsic mathematical property* of the Rasch model and one of its main requirements. Therefore, what is investigated is if the measurement of stress by the PSS-14 approximates the fundamental properties of measurement by satisfying the *a priori* measurement requirements of the Rasch Model. Therefore, when the measurement requirements encompassed in the Rasch Model are not achieved, it is the discrepancies that will inform problems with the items and the psychological instrument [62]. In summary, the items fitting a Rasch model exhibit the measurement properties of (1) unidimensionality: the items measure a single psychological trait (i.e. perceived stress); (2) monotonicity: endorsement of “higher” categories are an increasing function of the latent trait; (3) homogeneity: the item difficulty order is the same for all respondents; (4) local independence: items are conditionally independent given the latent trait; and (5) absence of differential item functioning: items are conditionally independent of exogenous variables given the latent trait [52, 58].

#### Statistical analysis

The Rasch analysis was conducted with the RUMM2030 software[63]. The person estimates scatterplot was adapted from Winsteps software [64]. Descriptive statistics and Agresti-Coull binomial 95% Confidence Intervals (C.I.) were computed with R software [65].

The first stage in the analysis was to determine model parametrization and investigate model fit. The estimation method used was Pairwise Conditional Maximum Likelihood Estimation. The advantage of Pairwise Conditional Maximum Likelihood Estimation over Conditional Maximum Likelihood Estimation is that the estimates of each item parameter is a function of frequencies in all categories rather than just a function of frequencies of the adjacent categories. Therefore, the estimates of item parameters are less affected by categories with a small frequency of responses [66]. The model was initially estimated with an unrestricted parametrization (i.e. Partial Credit model). However, considering that the distances between item thresholds might be equal across items (i.e. Rating Scale model) in certain cases, a likelihood-ratio test was conducted to determine whether an unrestricted parametrization (Partial Credit model) would provide a better description of the item responses in comparison with a restricted parametrization (Rating Scale model) [67, 68]. Missing data for individual items ranged from 0.0% to 1.1%, and missing was 1.2% when considering all items. The impact of missing values was unsubstantial [69] and a Rasch analysis with missing values has been shown to outperform multiple imputation under most conditions [70, 71]. Therefore, the Rasch analysis with missing values was conducted. Additionally, in the conditional framework, a sample size around 390 participants is sufficient to detect a model deviation in item difficulty of 1 logit with a significance level of 5% and power of 80%. However, since these samples requirements are influenced by several factors including targeting [72], other authors have argued that samples with more than 250 participants provide enough power for most practical purposes [73]. The overall model fit was evaluated with a summary item-trait χ^2^ statistic, which is calculated through the summation of the χ^2^ of all individual items [63].

The second stage was the analysis of dimensionality. The dimensionality was evaluated through a Principal Component Analysis (PCA) of the residuals. In this procedure, two item sets were identified according to their opposite loadings on the first residual factor. The persons’ estimates of these two item sets were compared through independent t-tests. If the data is unidimensional it is expected less than 5% of these differences to be statistically significant [74]. In addition, an Agresti-Coull binomial 95% CI was computed for the proportion of statistically significant differences [75, 76]. To investigate the strength of the association between the persons’ estimates, the disattenuated correlation coefficient was calculated [77]. When multidimensionality was detected, the Rasch model was applied to each subscale independently to assess validity and reliability. In addition, a subtest analysis was conducted to evaluate the magnitude of the multidimensionality through the estimation of the latent correlation between subscales. The latent correlation is a single effective summary about how the distinct latent traits are associated and is estimated based on the change in the reliability index when subscales are analysed separately compared to when they are analysed combined [63].

The third stage was the evaluation of response dependence [78]. Response dependence was evaluated through the correlation matrix of standardized residuals [79]. The magnitude of the observed residual correlations is considered *relative to the overall set of correlations*, and higher values *compared to the average residual correlation* indicate that the items responses were not completely accounted by the psychological trait. Therefore, it is also presented the adjusted residual correlations, which are the differences between the observed residual correlations and the average residual correlations. In addition, a cut-off point of 0.15 for the adjusted residual correlations from simulation studies that evaluated polytomous items in a sample size of 350 participants was used [80, 81]. Response dependence was resolved through the creation of composite items (i.e. “super items”). To create composite items, the individual items were summated [82, 83].

The following stage was the analysis of Differential Item Functioning [84]. In this study, the investigation of uniform and non-uniform DIF was conducted statistically, through a two-way ANOVA of the residuals [85] with the calculation of η^2^ and partial η^2^ as measures of effect size [86]; and graphically, by diving the sample based on the trait level into adjacent class interval (CI) and plotting the average observed item responses against the model theoretical expectations indicated by the Item Characteristic Curves (ICCs). Uniform DIF refers to when the magnitude of the conditional dependence between item responses and exogenous variable given the latent trait is constant across the trait level. When the magnitude of the conditional dependence between item responses and exogenous variable given the latent trait varies across the trait level, it is said that the item has non-uniform DIF [87]. Ideally, the sample would be divided into a unique group for each possible total score (and, therefore, person estimate), but since it is unlikely to have the necessary amount of respondents with the same total score for each possible score, class intervals (CI) were created [63, 88]. The characteristics analysed for DIF were age group (14 to 20 years old; 21 to 30 years old; 31 years old or more); education (education level up to High School; TRADE, TAFE, or University); socioeconomic position (1^st^, 2^nd^, 3^rd^, 4^th^ and 5^th^ quintiles of the IRSAD); and smoking status (Never smoked tobacco; Used to smoke tobacco; and Currently smoke tobacco). The choice of age and education was due to DIF of PSS items been previously reported [39, 40]. It was also hypothesized that socioeconomic position and smoking status could be additional sources of DIF. In case DIF was present, it was solved by splitting the item into group specific items [85]. Considering that in the analysis of DIF multiple null hypothesis significance testing (NHST) were performed, to avoid the increase in the probability of performing a Type 1 error, a Bonferroni adjustment with α = 0.01 was applied [89].

The fifth stage was the analysis of item threshold ordering [90]. It was investigated if the thresholds positions were ordered and if every category became the most probable for a definitive range of the latent trait. In case disordering was found, adjacent categories were considered to be collapsed [68]. The only exception was composite items since the disordering is a consequence of local dependence and collapsing categories is not necessary [58].

Item misfit was analysed only after evidence of unidimensionality, local independence, and absence of DIF was established. If these three conditions are violated, the idea that separate items should fit a Rasch model loses its meaning [61]. The assessment of fit in the Rasch Model was conducted using a “family approach” [91] through statistical and graphical evaluation of the Item Characteristic Curves. Statistically, the *magnitude of* item misfit was evaluated with the Fit Residual statistic, with the value of 0 meaning fit of the data to the Rasch model and acceptable values between -2.5 and 2.5 [63, 88]. Additionally, the χ^2^ statistic was used to evaluate item-trait interaction and the *probability* of the misfit occurring due to sampling variation [63, 85]. To consider an item as misfitting, the misfit needed to be flagged by both statistics [92] and a Bonferroni adjustment with α = 0.01 was applied [89]. Similarly to the DIF analysis, the graphical analysis was conducted by evaluating the average observed item responses in each CI compared to the expected item responses.

The subsequent stage of the analysis was the evaluation of targeting. The analysis investigated the mean of the persons’ parameters [76], and a value between ±0.5 logits indicates optimal targeting [93]. The internal consistency reliability was analysed with the Person Separation Index (PSI), an index analogous in construction to the Cronbach’s α [94] but calculated using the persons’ parameters rather than the raw scores.

The last stage of the analysis was the investigation of criterion validity. Criterion validity was evaluated by: a) inspecting convergent and divergent validity of the latent trait with theoretically relevant constructs of its nomological network [95]; and b) concurrent validity with respect to health behaviours. For the analysis of convergent and divergent validity, the selected constructs were sense of personal control and social support. It was expected a negative correlation of perceived stress with MS and SSS, and a positive correlation with PC. Since the PSS, SPCS and SSS scores are ordinal, the non-parametric Kendall’s τ was used [96]. A Bonferroni adjustment was applied for the calculation of 95% C.I. [89].

For the analysis of concurrent validity, the mean of the total scores for each subscale was used to dichotomize the participants into lower perceived distress/higher perceived distress and lower perceived coping/higher perceived coping. Log-binomial models were then used to evaluate if there was an effect of perceived distress (or perceived coping) on smoking status and alcohol consumption after controlling for the confounders general health and education. It was expected that participants with higher perceived distress would have a higher risk of currently smoking and drinking alcohol than participants with lower perceived distress, and participants with higher perceived coping would have a lower risk.

## Results

Model parametrization and model fit: The Likelihood Ratio test (LRT) showed that the model with an unrestricted parametrization was a significantly better fit to the data compared to the model with a restricted parametrization (χ^2^ (38) = 60.89, p = 0.011). Therefore, an unrestricted parametrization was chosen. After the model was estimated, the summary test-of-fit (χ^2^ (98) = 386.90, p < .001) indicated no overall fit to the Rasch Model.

Dimensionality: The analysis of dimensionality conducted through a PCA of the residuals (S1 Table) showed the positively worded and negatively worded items loading on the first residual factor with an opposite factor loading valence (S2 Table).

**Fig 1:**
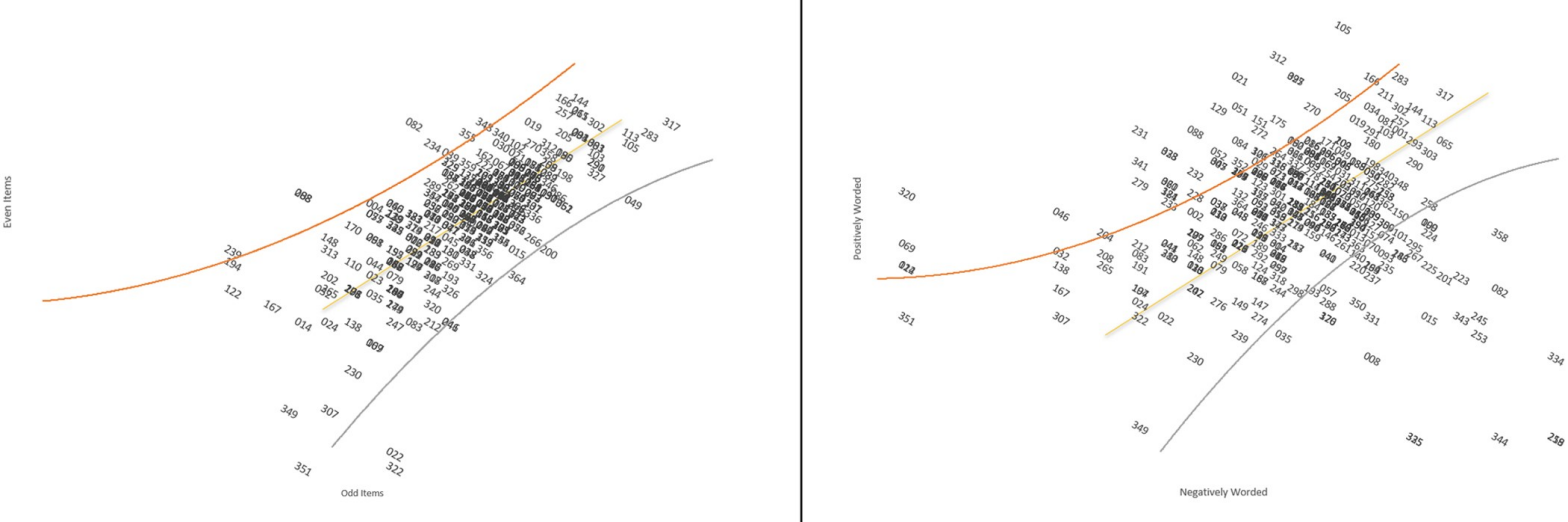
**Scatterplot of the persons estimates after the division of the items into even and odd (left) and positively worded and negatively worded items (right).** The de-identified numbers indicate the study participants. The x-axis and y-axis indicate the participants’ latent trait estimated from a different subset of items (e.g. in the picture on the left, the x-axis displays the participants’ latent trait according to their responses to odd-numbered items and the y-axis displays the participants’ latent trait according to their responses to even-numbered items). The yellow line is the identity line and the confidence bands are the 95% C.I.

The items were then divided into positively and negatively worded subsets and two estimates for each participant were calculated based on these two subsets of items. The estimates were then compared, which led to 21.7% statistically significant t-tests (95% Agresti-Coull C.I. [16.6%, 24.1%]) and a disattenuated correlation coefficient of 0.06. To illustrate the effects of multidimensionality, the items were also divided into subsets of even and odd items. For even versus odd items, there were 2.2% statistically significant t-tests between the person estimates (95% Agresti-Coull C.I. [1.0%, 4.3%]) and a disattenuated correlation coefficient of 1.0 (Fig 1).

Therefore, the 21.7% statistically significant t-tests between person estimates from each subset (95% Agresti-Coull C.I. [16.6%, 24.1%]) and the disattenuated correlation coefficient of 0.06 indicated two dimensions composed of negatively worded (“Perceived Distress”) and positively worded (“Perceived Coping”) items. The items were separated into the two subscales and analysed separately. The latent correlation between the Perceived Distress and Perceived Coping subscales was r = 0.14 and the score correlation between the Perceived Distress and Perceived Coping subscales was r = 0.13.

### Negatively worded items (“Perceived distress”)

Model parametrization and response dependence: The Likelihood Ratio test (LRT) showed that the model with an unrestricted parametrization was not a significantly better fit to the data compared to the model with a restricted parametrization (χ^2^ (17) = 20.85, p = 0.233). For this reason, the model with a restricted parametrization was estimated. The summary test-of-fit (χ^2^ (42) = 80.31, p<0.001) indicated no overall fit to the Rasch Model.

Table 2 shows the residual correlations matrix where there was response dependence between items 1 (“…felt upset because of something that happened?”), 2 (“…felt like you couldn’t control the important things in your life?”) and 3 (“…felt nervous or stressed?”), and between items 11 (“…felt angered because of things that happened outside of your control?”), 12 (“…found yourself thinking about all the things that you have to do?”) and 14 (“…felt troubles were piling up so high that you could not deal with them?”). The average residual correlation was -0.16. The adjusted residual correlations surpassed the bootstrapped cut-off point of 0.15. These items were combined into two composite items and the Rasch model with an unrestricted parametrization was applied. The summary test-of-fit (χ^2^ (21) = 11.74, p = 0.946) indicated overall fit to the Rasch Model.

**Table 2:** Residual correlations of the negatively worded items.

		Item 1	Item 2	Item 3	Item 8	Item 11	Item 12	Item 14
Item 1		1						
Item 2	Obs	**0.095**	1					
	Adj	**0.255**						
Item 3	Obs	**0.043**	**0.046**	1				
	Adj	**0.203**	**0.206**					
Item 8	Obs	-0.273	-0.238	-0.296	1			
	Adj	-0.113	-0.078	-0.136				
Item 11	Obs	-0.256	-0.307	-0.221	-0.156	1		
	Adj	-0.096	-0.147	-0.061	0.004			
Item 12	Obs	-0.253	-0.344	-0.309	-0.102	**0.003**	1	
	Adj	-0.093	-0.184	-0.149	0.058	**0.163**		
Item 14	Obs	-0.288	-0.199	-0.143	-0.134	**0.039**	-0.148	1
	Adj	-0.128	-0.039	0.017	0.026	**0.199**	0.012	

Note. The residual correlations matrix displays the observed correlation between item responses after the influence of the latent trait (“Perceived Distress”) was accounted by the model. It is also displayed the adjusted residual correlations, which are the differences between the observed residual correlations and the average residual correlation.

**Fig 2:**
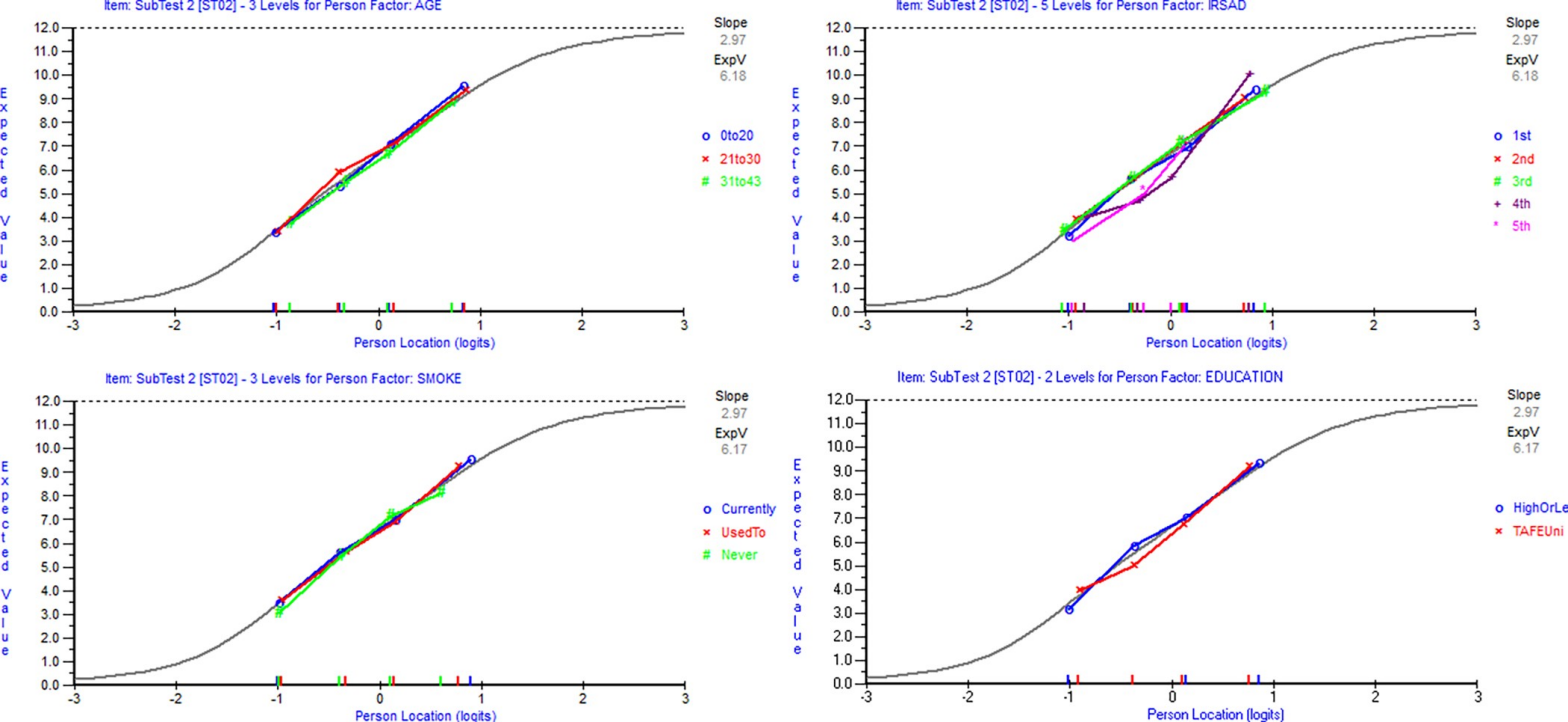
Analysis of Composite Item 2 DIF by Age (upper-left), Socioeconomic position (upper-right), Smoking status (bottom-left) and Education (bottom-right). Note. The colored points indicate the *average* observed item scores of each subgroup defined by an exogenous variable (e.g. the blue points in the bottom-left graph represent the average item scores of participants who currently smoked, the red points represent the average item scores of participants who used to smoke, and the green points represent the average item scores of participants who never smoked). The grey logistic curve indicates the expected item responses. The slope of the Item Characteristic Curve indicates the rate of change of the expected value with respect to the latent trait at the mid-point between the minimum and maximum scores.

Dimensionality and DIF: The PCA of the residuals indicated the first residual factor loadings for Composite Item 1, composed of Items 1, 2 and 3 (Λ = 0.988), Composite Item 2, composed of Items 11, 12 and 14 (Λ = -0.877) and Item 8 (Λ = -0.292). Composite Item 2 and Item 8 were combined into a subset, and it was found 5.85% statistically significant t-tests between the person estimates (95% Agresti-Coull C.I. [3.81%, 8.82%]) and a disattenuated correlation coefficient of 1.00, indicating insufficient evidence to support multidimensionality. No uniform and non-uniform DIF were found regarding age, socioeconomic position, education and smoking status (Fig 2) (S3 Table).

Threshold ordering: Composite Item 1 and 2 had disordered thresholds and Item 8 thresholds were ordered (S4 Table). Since only composite items had threshold disordering, collapsing categories was not necessary.

Item fit: The individual item fit statistics is displayed below (Table 3). A Bonferroni adjustment of 0.0033 for the 3 items was applied.

**Table 3:** The fit of the revised negatively worded items (“Perceived distress”) to the Rasch Model.

Item^i^	Location	SE	Fit Residual	*df*	*χ2*	*df*	Prob
Composite Item 1	-0.030	0.030	-0.958	230.58	4.514	7	0.299
Composite Item 2	-0.159	0.030	-2.118	233.19	3.92	7	0.828
8. felt unable to cope with all the things that you had to do?	0.189	0.063	**3.104**	231.23	3.304	7	0.378

i. Every item started with the sentence “How often during the LAST YEAR have you….”. Note. The second column displays the items’ location on the latent trait scale (i.e. the item difficulty). Values of the Fit Residual statistic indicating item misfit (i.e. lower than -2.5 or higher than 2.5), as well as statistically significant χ2 indicating misfit due to item-trait interaction, were highlighted in bold.

**Fig 3:**
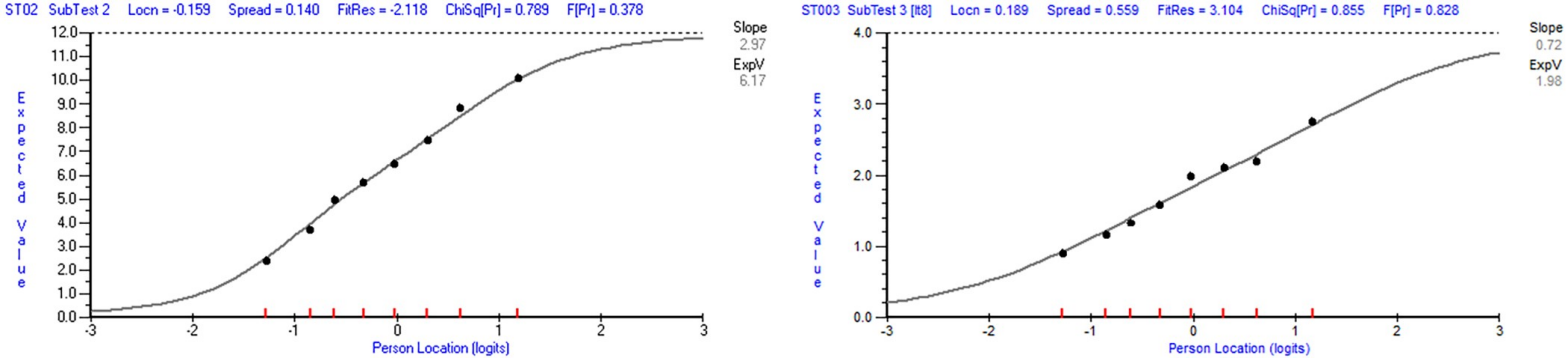
**Item characteristic curve for Composite Item 2 (left) and Item 8 (right).** Note. The x-axis indicates the latent trait and the y-axis indicates the item score. The black points represent the *average* observed item responses in each class interval. The grey logistic curve indicates the expected item responses. The slope of the Item Characteristic Curve indicates the rate of change of the expected value with respect to the latent trait at the mid-point between the minimum and maximum scores.

Item 8 (Fit Residual = 3.104; χ2 (7) = 3.304, p = 0.828) had a higher Fit Residuals but was not flagged as misfitting by the χ^2^-statistic. The graphical evaluation of the ICC (Fig 3) indicated that deviations of the average observed item responses in comparison to the expected item responses were unsubstantial. For these reasons, Item 8 was not considered as misfitting and was retained.

Since overall model fit was achieved and there was no more evidence of DIF, local dependence, thresholds disordering or item misfit, the measurement model for the negatively worded items (“Perceived Distress”) was established.

Targeting and reliability: The Perceived Distress subscale is well targeted for the Aboriginal pregnant women. The mean of perceived distress was -0.179 logits and item thresholds covered almost all of the persons’ distribution (S1 Fig). The PSI was 0.72, exhibiting a decrease when compared to the value of 0.82 before the creation of composite items.

### Positively worded items (“Perceived coping”)

Model parametrization: The Likelihood Ratio test (LRT) indicated that the model with an unrestricted parametrization was not a significantly better fit to the data compared to the model with a restricted parametrization (χ^2^ (17) = 11.14, p = 0.849). For this reason, the model with a restricted parametrization was chosen. The summary test-of-fit (χ^2^ (49) = 86.20, p = < .001) showed no overall fit to the Rasch Model.

Dimensionality: The PCA of the residuals showed that Item 4 (Λ = 0.725) and Item 5 (Λ = 0.537) had factor loadings with an opposite valence compared to the other items (S5 Table). After combining them into a subset and comparing with a subset composed of the other items, there were 7.71% statistically significant t-tests between the person estimates (95% Agresti-Coull C.I. [5.35%, 10.95%]) and a disattenuated correlation coefficient of 0.84. The PCA/t-test procedure indicated that item 4 and/or item 5 might have been measuring a different psychological trait, and the higher factor loading of item 4 suggested this item to be the most affected by multidimensionality.

Response dependence: The standardized residual correlation matrix (S6 Table) showed response dependence between items 5 (“…coped well with important changes in your life?) and 6 (“…felt able to handle your personal problems?), and between items 7 (“…felt things were going your way?) and 10 (“…felt you were on top of things?). The average residual correlation of positively worded items was also -0.16 and the adjusted residual correlations surpassed the bootstrapped cut-off point of 0.15.

**Fig 4:**
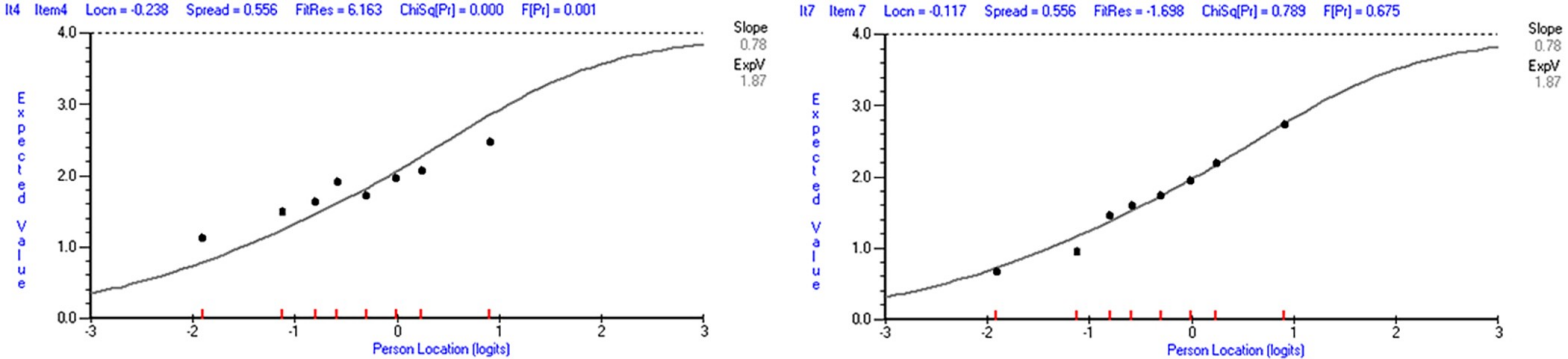
**Item characteristic curve for Item 4 (left) and Item 7 (right).** Note. The x-axis indicates the latent trait and the y-axis indicates the item score. The black points represent the *average* observed item responses in each class interval. The grey logistic curve indicates the expected item responses. The slope of the Item Characteristic Curve indicates the rate of change of the expected value with respect to the latent trait at the mid-point between the minimum and maximum scores.

DIF and Item fit: The analysis of DIF showed no uniform and non-uniform DIF by age, socioeconomic position, education and smoking status (S7 Table). However, misfit by item-trait interaction was consistently flagged for Item 4, when DIF was analysed by age (η^2^ = 0.111, η_p_^2^ = 0.112, p<0.001), socioeconomic position (η^2^ = 0.106, η_p_^2^ = 0.110, p<0.001), education (η^2^ = 0.106, η_p_^2^ = 0.107, p<0.001), and smoking status (η^2^ = 0.099, η_p_^2^ = 0.100, p<0.001). Finally, the analysis of item fit (S8 Table) also indicated misfit for item 4 (Fit Residual = 6.083; χ^2^ (7) = 52.751, p<0.001). Although the η_p_^2^ found according to Cohen’s [97] benchmarks for η_p_^2^ is a medium effect size, interpretation of what is a “medium” effect size differs according to the field, and the Fit Residual more clearly illustrates the magnitude of Item 4 misfit. The Item Characteristic Curve displayed a clear pattern of under discrimination (Fig 4).

The evidence that item 4 (“How often during the LAST YEAR have you dealt well with life hassles?”) was affected by multidimensionality, and the misfit could not be explained by response dependence or DIF, indicated that this item was not a good measure of perceived coping and lead to its deletion.

### Analysis of the revised positively worded items (“Perceived Coping”)

Model fit and dimensionality: After the exclusion of item 4, items 5 and 6, and item 7 and 10 were combined into two composite items. The unrestricted parametrization was applied and the new model achieved fit to the Rasch model (χ^2^ (28) = 17.63, p = 0.935). The PCA of the residuals showed the factor loadings of Composite Item 3, composed of items 5 and 6 (Λ = 0.894), Composite Item 4, composed of items 7 and 10 (Λ = -0.827), item 9 (Λ = 0.015) and item 13 (Λ = -0.112) to the first residual factor. Composite Item 3 and item 9 were combined into a subset, while Composite Item 4 and item 13 were combined into another subset. It was found 4.13% statistically significant t-tests between the person estimates (95% Agresti-Coull C.I. [2.47%, 6.76%]) and a disattenuated correlation coefficient of 1.00. In summary, after the deletion of Item 4, there was no more evidence of multidimensionality.

Threshold ordering: The new analysis indicated also that the upper thresholds for Item 13 (“…felt able to control how you spend your time?) were disordered (Table 4) (S2 Fig).

**Table 4:** Threshold ordering of the positively worded items.

	Composite Item 3	SE	Composite Item 4	SE	Item 9	SE	Item 13	SE
Threshold 1	-1.911	0.164	-2.194	0.201	-2.350	0.124	-1.579	0.124
Threshold 2	-1.525	0.140	-1.580	0.154	-0.315	0.113	-0.635	0.114
Threshold 3	-0.939	0.133	-0.905	0.134	1.076	0.146	**1.234**	0.167
Threshold 4	-0.249	0.146	-0.213	0.136	1.590	0.209	**0.981**	0.212
Threshold 5	0.449	0.187	0.453	0.157				
Threshold 6	1.059	0.272	1.049	0.205				
Threshold 7	1.485	0.414	1.532	0.288				
Threshold 8	1.630	0.581	1.858	0.407				

Note. The item threshold parameter indicates on the latent trait scale the point in which there is an equal probability of response to adjacent categories. Items disordered thresholds are highlighted in bold. The last two thresholds of Item 13 are disordered since the equal probability of selecting the categories “Very often” or “Fairly often” occur for participants with lower Perceived Coping (0.981 logits) than the equal probability of selecting the categories “Sometimes” or “Fairly often” (1.234 logits).

Collapsing the categories of “Fairly often” with “Sometimes” (χ^2^ (49) = 540.46, p = < .001), or “Fairly often” with “Very often” (χ^2^ (49) = 282.99, p = < .001) disturbed the measurement model. Since collapsing was not possible, the deletion of item 13 was considered. However, the benefits of retaining item 13 in terms of content validity were considered greater than the benefits of excluding it. Notwithstanding, the ordering of item 13 thresholds should be further investigated in future studies with an Aboriginal population.

Item fit: For the analysis of item fit, a Bonferroni adjustment of 0.0025 for the 4 items was applied and no misfit was found (S9 Table). The overall fit to the Rasch model, combined with no further evidence of DIF, local dependence, and item misfit, lead to the establishment of the measurement model for the positively worded items.

Targeting and reliability: The Perceived Coping subscale is well targeted for the Aboriginal pregnant women, with the mean of perceived coping of -0.363 logits (S3 Fig). The PSI was 0.76, a smaller value when compared to 0.80 before the creation of the composite items.

### Dimensionality of the revised scales

After the establishment of the two measurement models, a final dimensionality analysis was conducted to test if, after response dependence was accounted with composite items, the revised PSS-14 would exhibit a unidimensional structure. The items 8, 9 and item 13, and composite items 1, 2, 3 and 4 were modeled together and the PCA/t-test procedure applied. The negatively worded (Composite Item 1, Composite Item 2 and Item 8) had positive loadings, while the positively worded (Composite Item 3, Composite Item 4, Item 9 and Item 13) had negative loadings on the first residual factor (S10 Table). It was found 15.4% statistically significant t-tests between the person estimates (95% Agresti-Coull C.I. [12.0%, 19.5%]) and a disattenuated correlation coefficient of 0.25. After the creation of composite items, the revised PSS-14 still exhibits a two-factor structure.

### Criterion validity

The subscales of Perceived Distress and Perceived Coping displayed the expected patterns of convergence and divergence regarding Mastery, Perceived Constraints and Social Support (Table 5). The only exception was the relationship between Perceived Distress and Mastery, which exhibited a weak non-significant correlation (r = -0.13 95% C.I. [-0.29, 0.05]).

**Table 5:** Correlations between Perceived Distress and Perceived Coping subscales with Mastery, Perceived Constraints and Social Support.

	Mastery	95% C.I	Perceived Constraints	95% C.I	Social Support Scale	95% C.I
PSS subscales						
Perceived Distress	**-0.13**	**[-0.28, 0.02]**	0.29	[0.14, 0.42]	-0.21	[-0.35, -0.06]
Perceived Coping	0.33	[0.19, 0.45]	-0.32	[-0.45, -0.18]	0.28	[0.13, 0.41]

Note. The table displays the Kendall’s τ rank correlations and 95% CI between total (i.e. summated) scores of the Perceived Distress and Perceived Coping subscales and other psychological instruments that evaluate constructs pertaining to the Perceived Stress’ nomological network.

The analysis of concurrent validity indicated that participants with higher perceived coping had a 10% lower risk of smoking (RR = 0.90–95% C.I. [0.73, 1.10]) and a 31% lower risk of drinking alcohol (RR = 0.69–95% C.I. [0.35, 1.36]) compared to participants with lower perceived coping. In addition, participants with higher perceived stress had a 24% higher risk of smoking (RR = 1.24–95% C.I. [1.01, 1.52]) but no effect was found on alcohol consumption (RR = 0.99–95% C.I. [0.52, 1.90]).

The score distribution of the revised PSS-14 is displayed in S11 Table.

## Discussion

The aim of this study was to evaluate if the PSS-14 constitutes a valid and reliable instrument to measure perceived stress in an Aboriginal population. The results indicated that revised versions of the negatively worded (“Perceived Distress”) and positively worded (“Perceived Coping”) subscales are valid and modestly reliable for an Aboriginal population. However, the relationship between these two psychological traits is not meaningful and a total score of all items should not be created.

The present study started by investigating the main source of debate regarding the PSS, the dimensionality of the scale. The results are consistent with the literature about the PSS-14 indicating a two-factor structure [31]. When the two subscales were analysed separately, there was evidence of local dependence among items. For example, the response dependence between items 7 (“…felt things were going your way?) and 10 (“…felt you were on top of things?) is a form of redundancy-dependency. The overlap of item content works as if the same question was asked twice, but with a slightly different wording [80]. In this case, although these two items are similar enough to measure the latent trait of perceived coping, they are not different enough to produce one item worth of new information [98]. Since reliability indices such as Cronbach’s α and PSI are computed under the assumption that items are conditionally independent, these indices can be inflated in the presence of locally dependent items [99]. This phenomenon was observed in the current study since after the local dependence was accounted through the creation of composite items, there was a reduction of the PSI for both subscales.

The internal consistency reliability of the revised PSS-14 subscales (PSI_distress_ = 0.72; PSI_coping_ = 0.76) for a population of pregnant Aboriginal women was only modest [100]. Future applications of the revised PSS-14 in an Aboriginal population should consider that, although reliability between .70 and .80 is usually deemed adequate for research purposes [101], it does not provide enough measurement precision to be applied in high-stake scenarios [102], such as the use of test results to inform interventions (e.g. which participants should receive an intervention to reduce stress or stop receiving an intervention due to stress). Therefore, the inclusion of new locally independent to increase reliability is recommended. For example, Larson et al. [103] discuss the impact of racism in Aboriginal Australians and suggests the item “Within the past for weeks, have you experienced any physical stress or symptoms as a result of how you were treated because of your race?”.

A possible explanation for the modest reliability is that, despite both subscales being well-targeted, the levels of perceived stress (*M* = -0.18, *SD* = 0.75) and perceived coping (*M* = -0.36, *SD* = 1.10) (S1 Fig) (S3 Fig) were homogenous across the respondents. Aboriginal and Torres Strait Islander comprise a heterogeneous and culturally diverse population [104] but the social inequalities resulting from the process of colonization and subsequent marginalization were experienced *as a group* [105]. Therefore, it is unsurprising that levels of perceived stress (and perceived coping to a lesser extent) did not have a high variation (i.e. high standard deviation) in this group. Future studies should further investigate the reliability of the revised PSS-14 in other Aboriginal samples (i.e. more heterogeneous, containing men, wider age range, etc) and the previous recommendations based on reliability should be taken only as guidelines.

One finding of this study is that the variance of item responses of the PSS-14 could not be explained by the two psychological traits *or* by response dependence among items alone. The variance of the item responses was explained by both the two psychological traits *and* response dependence. The results contrast with findings from Medvedev, Krägeloh [38] about the PSS-10, in which after composite items were created to account for response dependence, the scale exhibited a unidimensional structure (5.2%–95% C.I. [3.2%, n.a]). In our study, the evidence of multidimensionality was clear (15.4%–95% Agresti-Coull C.I. [12.0%, 19.5%]) even after the inclusion of composite items.

The main finding of this study was the relationship between Perceived Distress and Perceived Coping subscales (r = 0.14). The low latent correlation contrasts with the overall literature about the PSS, in which latent factor correlations usually range from r = 0.50 to r = 0.70 [106], sometimes reaching values (>0.80) that pose a threat to discriminant validity [107, 108]. The plausible explanation for the low latent correlation between subscales (r = 0.14) is that the social inequalities experienced by the Aboriginal population are so pronounced that even Aboriginal pregnant women that perceived themselves as coping well with life challenges ended up endorsing items regarding high levels of stress. This means that, irrespectively of their behavioral coping abilities, the Aboriginal pregnant women could not escape experiencing stressful life events. This plausible explanation was further supported by the weak non-significant relationship between Perceived Distress and Mastery (r = -0.13 95% C.I. [-0.29, 0.05]). In contrast to Brown et al. [1], the perception of stress by the Aboriginal mothers were largely unrelated to Mastery, their beliefs regarding the ability to influence external outcomes.

The weak association between Perceived Distress and Perceived Coping/Mastery in Aboriginal Australians is congruent with Lazarus’ [26] theory of stress and coping. The negative association between Perceived Stress and Perceived Coping/Mastery in the general population exists because, even if *an event is appraised as threatening*, in case the individual has *a perception of sufficient coping resources* to *influence the event’s outcomes*, the stress reaction will be attenuated. However, the hypothesis is that in the case of Aboriginal Australians the frequency of stressful events is so disproportionately high that individual coping capabilities (perceived coping) and the ability to influence outcomes (mastery) are not enough to deal with the increasing amount of problems. Therefore, the relationship between coping, mastery and stress is weakened. It should be noticed that the relationship between Perceived Coping and Mastery (r = 0.33 95% C.I. [0.17, 0.47]) *was not affected* and worked as expected. The weak association between Perceived Stress and Perceived Coping/Mastery indicates that perceived stress nomological network might have shifted [95]. For this reason, a composite score for Perceived Stress should not be created and both Perceived Distress and Perceived Coping scales must be analysed separately.

When the associations of the Perceived Distress and Perceived Coping subscales with health behaviors were investigated, the expected relationships were also found. Higher perceived coping was found to reduce the risk of smoking and drinking alcohol, while higher perceived distress was found to increase the risk of smoking. Despite the exception that no effect was found of perceived distress on alcohol consumption, these findings provide further support that the revised PSS-14 is construct valid for this population.

Finally, the sample had a mean score of 13.35 (SD = 5.24) on the Perceived Distress subscale and a mean score of 9.82 (SD = 4.40) on the Perceived Coping subscale. Although the score distribution (S11 Table) can be used to distinguish stress levels between individuals, the interpretation of mean scores in terms of how much stress is experienced by this Aboriginal population is not simple. Firstly, there is an almost absence of cut-offs in the literature. A few recent studies have proposed cut-off points for the PSS-14 to distinguish between low-stress and high-stress respondents. For example, a total score equal or higher to 28 has been used to define “stressed” as opposed to “non-stressed” in India [109, 110] and Pakistan [111]. However, this proposed cut-off is for total scores summed across all items (PSS-14), whether the findings of our study indicated that for Aboriginal Australians the total scores of each subscale should be used independently. In addition, Cohen and the original authors argued that the PSS is not a diagnostic instrument, so there should be no cut-offs for “high”, “medium” or “low” stress and comparisons must be conducted within the sample [112], a position which became a general consensus in the PSS literature [113, 114]. Secondly, it is necessary first to ensure measurement invariance across culture, otherwise, the comparison of scores (or the use of cut-off points derived in another culture) is subject to cultural bias. Future studies should evaluate whether the revised PSS-14 is *cross-culturally valid* to inform the amount of perceived stress in an Aboriginal population by comparing it to a distinct cultural group.

There is a clear limitation of the present study. The study sample was composed of Aboriginal pregnant woman and, although no DIF was found regarding age, socioeconomic position, education, and smoking status, it was not possible to evaluated DIF by gender. Future studies should be conducted to investigate if the functioning of the revised PSS-14 is equivalent for men and non-pregnant Aboriginal women. In addition, when evaluating DIF, some subgroups had small sample sizes and it is not clear whether there was sufficient power to detect true differences. It is recommended future studies of the PSS-14 in a larger Aboriginal sample, which will increase the power to detect not only DIF but other deviations from the model. Finally, only concurrent validity was evaluated, so exposure and outcomes were measured simultaneously. Future studies should also examine predictive validity, with health behaviors (such as smoking status and alcohol consumption) measured at a second-time point, to allow inference regarding causality.

## Conclusions

One of the groups most at risk of stress in Australia is pregnant Aboriginal women. The present research showed that a revised PSS-14 is a culturally valid and modestly reliable psychological instrument to measure stress in an Aboriginal population. The relationship between the Perceived Distress and Perceived Coping subscales was weak and the two psychological traits need to be evaluated separately (i.e. a total score across all items should not be created). The use of the revised PSS-14 can provide culturally valid measurement of stress and inform future health research to better understand the role of stress in Aboriginal and Torres Strait Islanders well-being.

## Supporting information

S1 Table Principal Component analysis of the residuals. Note. The table displays the principal components of the item responses’ residuals, the eigenvalues of each component, the percentage of total explained variance and the standard errors.
(DOCX)

S2 Table Factor loadings on the first Principal Component. i. The items 4, 5, 6, 7, 9, 10 and 13 constituted the positively worded items. Note. The table displays the factor loadings of the items responses’ residuals on the first principal component (i.e. the first residual component).
(DOCX)

S3 Table Analysis of DIF of the negatively worded items. i. For each of the 9 NSHT, a Bonferroni adjustment of 0.0055 was applied. The table displays the results of the two-way ANOVA of the residuals according to class intervals (i.e. item-trait interaction) on the first four columns; according to subgroups defined by the exogenous variables (i.e. uniform DIF) on the next four columns; and according to the interaction between exogenous variables and class intervals (i.e. non-uniform DIF) on the last four columns. Statistically significant p-values are highlight in bold.
(DOCX)

S4 Table Threshold ordering of the negatively worded items. Note. The item threshold parameter indicates on the latent trait scale the point in which there is equal probability of response to adjacent categories. Items disordered thresholds are highlighted in bold.
(DOCX)

S5 Table Factor loadings on the first Principal Component of the positively worded items. Note. The table displays the factor loadings of the items responses’ residuals on the first principal component (i.e. the first residual component).
(DOCX)

S6 Table Residual correlations of the positively worded items. Note. The residual correlations matrix displays the observed correlation between item responses after the influence of the latent trait (“Perceived Coping”) was accounted by the model. It is also displayed the adjusted residual correlations, which are the differences between the observed residual correlations and the average residual correlation.
(DOCX)

S7 Table Analysis of DIF of the positively worded items. i. For each of the 21 NSHT, a Bonferroni adjustment of 0.0024 was applied. Note. The table displays the results of the two-way ANOVA of the residuals according to class intervals (i.e. item-trait interaction) on the first four columns; according to subgroups defined by the exogenous variables (i.e. uniform DIF) on the next four columns; and according to the interaction between exogenous variables and class intervals (i.e. non-uniform DIF) on the last four columns. Statistically significant p-values are highlight in bold.
(DOCX)

S8 Table The fit of the positively worded items (“Perceived coping”) to the Rasch Model. i. Every item started with the sentence “How often during the LAST YEAR have you….”. Note. The second column displays the items’ location on the latent trait scale (i.e. the item difficulty). Values of the Fit Residual statistic indicating item misfit (i.e. lower than -2.5 or higher than 2.5), as well as statistically significant χ2 indicating misfit due to item-trait interaction, were highlighted in bold.
(DOCX)

S9 Table The fit of the revised positively worded items (“Perceived coping”) to the Rasch Model. i. Every item started with the sentence “How often during the LAST YEAR have you….”. Note. The second column displays the items’ location on the latent trait scale (i.e. the item difficulty). Values of the Fit Residual statistic indicating item misfit (i.e. lower than -2.5 or higher than 2.5), as well as statistically significant χ2 indicating misfit due to item-trait interaction, were highlighted in bold.
(DOCX)

S10 Table Factor loadings on the first Principal Component of the two revised scales. Note. The table displays the factor loadings of the items responses’ residuals on the first principal component (i.e. the first residual component).
(DOCX)

S11 Table Score distribution of the revised PSS-14. Note. The table displays the score distribution of the revised Perceived Distress and Perceived Coping subscales. The items were responded on a five-point Likert scale (0 = Not at all, 1 = Rarely, 2 = Sometimes, 3 = Fairly often, 4 = Very often).
(DOCX)

S1 Fig Person-Item Threshold Map of the revised negatively worded items with the information function plotted in the background. Note. The top bars indicate the distribution of the persons parameters (i.e. “Perceived Distress”) and the bottom bars indicate the distribution of the item thresholds. The Fisher Information function is plotted on the background (i.e. the green line). It should be noticed that the distribution of item thresholds matches the distribution of person parameters throughout the latent trait indicating good targeting of the revised Perceived Distress subscale for this population.
(TIFF)

S2 Fig **Categories probability curves of Item 9 (left) and Item 13 (right)**. Note. The graph indicates the probability of endorsing a category according to the latent trait. It can be noticed that for Item 13 category 3 (“Fairly often”) never became the most probable for any range of the latent trait scale.
(TIFF)

S3 Fig Person-Item Threshold Map of the revised positively worded items with the information function plotted in the background. Note. The top bars indicate the distribution of the persons parameters (i.e. “Perceived Coping”) and the bottom bars indicate the distribution of the item thresholds. The Fisher Information function is plotted on the background (i.e. the green line). It can be notice that the distribution of person parameters throughout the latent trait is matched with the distribution of item thresholds indicating good targeting for this population. It should be noticed that the distribution of item thresholds matches the distribution of person parameters throughout the latent trait indicating good targeting of the revised Perceived Coping subscale for this population.
(TIFF)
