# Supplementary material for: Stress beyond coping? A Rasch analysis of the Perceived Stress Scale (PSS-14) in an Aboriginal population
Source: PLoS One. 2019 May 3;14(5):e0216333. doi: 10.1371/journal.pone.0216333 (PMC6499425; doi:10.1371/journal.pone.0216333)
Supplement: S1 Table — Note. The table displays the principal components of the item responses’ residuals, the eigenvalues of each component, the percentage of total explained variance and the standard errors. (DOCX) [file pone.0216333.s001.docx]

# S1 Table.

| Principal Component | Eigenvalue | Percentage of explained variance | | SE |
| --- | --- | --- | --- | --- |
| PC1 | 4.018 | 28.70% | 0.563 | |
| PC2 | 1.522 | 10.87% | 0.204 | |
| PC3 | 1.290 | 9.21% | 0.171 | |
| PC4 | 1.086 | 7.76% | 0.139 | |
| PC5 | 1.001 | 7.15% | 0.130 | |
| PC6 | 0.783 | 5.59% | 0.098 | |
| PC7 | 0.759 | 5.42% | 0.105 | |
| PC8 | 0.693 | 4.95% | 0.097 | |
| PC9 | 0.688 | 4.91% | 0.094 | |
| PC10 | 0.594 | 4.24% | 0.088 | |
| PC11 | 0.574 | 4.10% | 0.087 | |
| PC12 | 0.511 | 3.65% | 0.080 | |
| PC13 | 0.479 | 3.42% | 0.075 | |
| PC14 | 0.002 | 0.02% | 0.053 | |
