# Supplementary material for: Stress beyond coping? A Rasch analysis of the Perceived Stress Scale (PSS-14) in an Aboriginal population
Source: PLoS One. 2019 May 3;14(5):e0216333. doi: 10.1371/journal.pone.0216333 (PMC6499425; doi:10.1371/journal.pone.0216333)
Supplement: S2 Table — i. The items 4, 5, 6, 7, 9, 10 and 13 constituted the positively worded items. Note. The table displays the factor loadings of the items responses’ residuals on the first principal component (i.e. the first residual component). (DOCX) [file pone.0216333.s002.docx]

**S2 Table.**

| Item | Item4 ^i^ | | Item5 | Item6 | Item7 | | Item9 | | Item10 | | Item13 | | Item1 | Item2 | | Item3 | | Item8 | | Item11 | | Item12 | | Item14 | |
| --- | --- | --- | --- | --- | --- | --- | --- | --- | --- | --- | --- | --- | --- | --- | --- | --- | --- | --- | --- | --- | --- | --- | --- | --- | --- |
| Factor Loading | -0.481 | -0.539 | | -0.593 | | -0.437 | | -0.557 | | -0.488 | | -0.590 | 0.565 | | 0.476 | | 0.573 | | 0.361 | | 0.666 | | 0.602 | | 0.508 |
