# Supplementary material for: Stress beyond coping? A Rasch analysis of the Perceived Stress Scale (PSS-14) in an Aboriginal population
Source: PLoS One. 2019 May 3;14(5):e0216333. doi: 10.1371/journal.pone.0216333 (PMC6499425; doi:10.1371/journal.pone.0216333)
Supplement: S3 Table — i. For each of the 9 NSHT, a Bonferroni adjustment of 0.0055 was applied. The table displays the results of the two-way ANOVA of the residuals according to class intervals (i.e. item-trait interaction) on the first four columns; according to subgroups defined by the exogenous variables (i.e. uniform DIF) on the next four columns; and according to the interaction between exogenous variables and class intervals (i.e. non-uniform DIF) on the last four columns. Statistically significant p-values are highlight in bold. (DOCX) [file pone.0216333.s003.docx]

**S3 Table.**

|  | Perceived Stress (Class Interval) | | | | Age^i^ | | | | Perceived Stress-by-Age | | | |
| --- | --- | --- | --- | --- | --- | --- | --- | --- | --- | --- | --- | --- |
|  | η2 | η_p_2 | *df* | Prob | η2 | η_p_2 | *df* | Prob | η2 | η_p_2 | *df* | Prob |
| Composite Item1 | 0.007 | 0.007 | 3 | 0.519 | 0.005 | 0.005 | 2 | 0.460 | 0.014 | 0.014 | 6 | 0.595 |
| Composite Item2 | 0.013 | 0.014 | 3 | 0.209 | 0.007 | 0.007 | 2 | 0.309 | 0.014 | 0.014 | 6 | 0.571 |
| Item 8 | 0.008 | 0.009 | 3 | 0.420 | 0.001 | 0.001 | 2 | 0.845 | 0.022 | 0.022 | 6 | 0.292 |
|  | Perceived Stress (Class Interval) | | | | Socioeconomic position | | | | Perceived Stress-by-Socioeconomic position | | | |
|  | η2 | η_p_2 | *df* | Prob | η2 | η_p_2 | *df* | Prob | η2 | η_p_2 | *df* | Prob |
| Composite Item1 | 0.005 | 0.005 | 3 | 0.611 | 0.021 | 0.021 | 4 | 0.130 | 0.013 | 0.014 | 11 | 0.946 |
| Composite Item2 | 0.015 | 0.015 | 3 | 0.152 | 0.013 | 0.014 | 4 | 0.317 | 0.025 | 0.026 | 11 | 0.624 |
| Item 8 | 0.005 | 0.005 | 3 | 0.623 | 0.006 | 0.006 | 4 | 0.703 | 0.050 | 0.051 | 11 | 0.091 |
|  | Perceived Stress (Class Interval) | | | | Education | | | | Perceived Stress-by-Education | | | |
|  | η2 | η_p_2 | *df* | Prob | η2 | η_p_2 | *df* | Prob | η2 | η_p_2 | *df* | Prob |
| Composite Item1 | 0.005 | 0.005 | 3 | 0.626 | 0.004 | 0.004 | 1 | 0.229 | 0.011 | 0.011 | 3 | 0.264 |
| Composite Item2 | 0.014 | 0.015 | 3 | 0.154 | 0.002 | 0.002 | 1 | 0.447 | 0.030 | 0.030 | 3 | 0.013 |
| Item 8 | 0.005 | 0.005 | 3 | 0.656 | 0.001 | 0.001 | 1 | 0.511 | 0.006 | 0.006 | 3 | 0.524 |
|  | Perceived Stress (Class Interval) | | | | Smoking Status | | | | Perceived Stress-by-Smoking Status | | | |
|  | η2 | η_p_2 | *df* | Prob | η2 | η_p_2 | *df* | Prob | η2 | η_p_2 | *df* | Prob |
| Composite Item1 | 0.005 | 0.006 | 3 | 0.594 | 0.004 | 0.004 | 2 | 0.539 | 0.012 | 0.012 | 6 | 0.645 |
| Composite Item2 | 0.014 | 0.014 | 3 | 0.170 | 0.006 | 0.007 | 2 | 0.319 | 0.010 | 0.010 | 6 | 0.726 |
| Item 8 | 0.004 | 0.005 | 3 | 0.672 | 0.017 | 0.018 | 2 | 0.049 | 0.008 | 0.008 | 6 | 0.846 |
