# Supplementary material for: Stress beyond coping? A Rasch analysis of the Perceived Stress Scale (PSS-14) in an Aboriginal population
Source: PLoS One. 2019 May 3;14(5):e0216333. doi: 10.1371/journal.pone.0216333 (PMC6499425; doi:10.1371/journal.pone.0216333)
Supplement: S4 Table — Note. The item threshold parameter indicates on the latent trait scale the point in which there is equal probability of response to adjacent categories. Items disordered thresholds are highlighted in bold. (DOCX) [file pone.0216333.s004.docx]

**S4 Table.**

|  | Composite Item 1 | SE | Composite Item 2 | SE | Item 8 | SE |
| --- | --- | --- | --- | --- | --- | --- |
| Threshold 1 | -0.703 | 0.281 | -1.196 | 0.323 | -1.581 | 0.124 |
| Threshold 2 | -1.050 | 0.254 | -1.223 | 0.267 | -0.959 | 0.108 |
| Threshold 3 | -1.114 | 0.211 | -1.116 | 0.218 | 1.076 | 0.158 |
| Threshold 4 | -0.958 | 0.175 | -0.902 | 0.182 | 1.465 | 0.268 |
| Threshold 5 | -0.644 | 0.154 | -0.609 | 0.161 |  |  |
| Threshold 6 | -0.234 | 0.150 | -0.262 | 0.152 |  |  |
| Threshold 7 | 0.209 | 0.161 | 0.111 | 0.155 |  |  |
| Threshold 8 | 0.623 | 0.190 | 0.484 | 0.171 |  |  |
| Threshold 9 | 0.946 | 0.239 | 0.829 | 0.200 |  |  |
| Threshold 10 | 1.115 | 0.301 | 1.120 | 0.245 |  |  |
| Threshold 11 | 1.068 | 0.345 | 1.330 | 0.304 |  |  |
| Threshold 12 | 0.742 | 0.332 | 1.432 | 0.368 |  |  |
