# Supplementary material for: Stress beyond coping? A Rasch analysis of the Perceived Stress Scale (PSS-14) in an Aboriginal population
Source: PLoS One. 2019 May 3;14(5):e0216333. doi: 10.1371/journal.pone.0216333 (PMC6499425; doi:10.1371/journal.pone.0216333)
Supplement: S6 Table — Note. The residual correlations matrix displays the observed correlation between item responses after the influence of the latent trait (“Perceived Coping”) was accounted by the model. It is also displayed the adjusted residual correlations, which are the differences between the observed residual correlations and the average residual correlation. (DOCX) [file pone.0216333.s006.docx]

**S6 Table.**

|  |  | Item 4 | Item 5 | Item 6 | Item 7 | Item 9 | Item 10 | Item 13 |
| --- | --- | --- | --- | --- | --- | --- | --- | --- |
| Item 4 |  | 1 |  |  |  |  |  |  |
| Item 5 | Obs | -0.057 | 1 |  |  |  |  |  |
|  | Adj | 0.103 |  |  |  |  |  |  |
| Item 6 | Obs | -0.324 | **0.058** | 1 |  |  |  |  |
|  | Adj | -0.164 | **0.218** |  |  |  |  |  |
| Item 7 | Obs | -0.277 | -0.194 | -0.162 | 1 |  |  |  |
|  | Adj | -0.117 | -0.034 | -0.002 |  |  |  |  |
| Item 9 | Obs | -0.262 | -0.199 | -0.132 | -0.188 | 1 |  |  |
|  | Adj | -0.100 | -0.039 | 0.028 | -0.028 |  |  |  |
| Item 10 | Obs | -0.308 | -0.245 | -0.093 | **0.086** | -0.063 | 1 |  |
|  | Adj | -0.148 | -0.085 | 0.067 | **0.246** | 0.097 |  |  |
| Item 13 | Obs | -0.190 | -0.294 | -0.100 | -0.138 | -0.116 | -0.184 | 1 |
|  | Adj | -0.030 | -0.134 | 0.060 | 0.022 | 0.044 | -0.024 |  |
