# Supplementary material for: Stress beyond coping? A Rasch analysis of the Perceived Stress Scale (PSS-14) in an Aboriginal population
Source: PLoS One. 2019 May 3;14(5):e0216333. doi: 10.1371/journal.pone.0216333 (PMC6499425; doi:10.1371/journal.pone.0216333)
Supplement: S7 Table — i. For each of the 21 NSHT, a Bonferroni adjustment of 0.0024 was applied. Note. The table displays the results of the two-way ANOVA of the residuals according to class intervals (i.e. item-trait interaction) on the first four columns; according to subgroups defined by the exogenous variables (i.e. uniform DIF) on the next four columns; and according to the interaction between exogenous variables and class intervals (i.e. non-uniform DIF) on the last four columns. Statistically significant p-values are highlight in bold. (DOCX) [file pone.0216333.s007.docx]

**S7 Table.**

|  | Perceived Stress (Class Interval) | | | | Age^i^ | | | | Perceived Stress-by-Age | | | |
| --- | --- | --- | --- | --- | --- | --- | --- | --- | --- | --- | --- | --- |
|  | η2 | η_p_2 | *df* | Prob | η2 | η_p_2 | *df* | Prob | η2 | η_p_2 | *df* | Prob |
| Item 4 | 0.111 | 0.112 | 3 | **0.000** | 0.004 | 0.005 | 2 | 0.495 | 0.001 | 0.006 | 6 | 0.923 |
| Item 5 | 0.051 | 0.053 | 3 | 0.008 | 0.008 | 0.009 | 2 | 0.253 | 0.006 | 0.039 | 6 | 0.060 |
| Item 6 | 0.053 | 0.054 | 3 | 0.016 | 0.002 | 0.002 | 2 | 0.783 | 0.002 | 0.015 | 6 | 0.595 |
| Item 7 | 0.034 | 0.034 | 3 | 0.013 | 0.000 | 0.000 | 2 | 0.937 | 0.002 | 0.011 | 6 | 0.774 |
| Item 9 | 0.018 | 0.019 | 3 | 0.593 | 0.001 | 0.001 | 2 | 0.862 | 0.004 | 0.027 | 6 | 0.226 |
| Item 10 | 0.054 | 0.054 | 3 | 0.004 | 0.003 | 0.004 | 2 | 0.577 | 0.002 | 0.012 | 6 | 0.705 |
| Item 13 | 0.031 | 0.032 | 3 | 0.263 | 0.003 | 0.003 | 2 | 0.658 | 0.003 | 0.019 | 6 | 0.450 |
|  | Perceived Stress (Class Interval) | | | | Socioeconomic position | | | | Perceived Stress-by-Socioeconomic position | | | |
|  | η2 | η_p_2 | *df* | Prob | η2 | η_p_2 | *df* | Prob | η2 | η_p_2 | *df* | Prob |
| Item 4 | 0.106 | 0.110 | 3 | **0.000** | 0.010 | 0.011 | 4 | 0.438 | 0.026 | 0.030 | 11 | 0.502 |
| Item 5 | 0.045 | 0.047 | 3 | 0.010 | 0.002 | 0.003 | 4 | 0.926 | 0.031 | 0.032 | 11 | 0.423 |
| Item 6 | 0.047 | 0.049 | 3 | 0.031 | 0.003 | 0.003 | 4 | 0.902 | 0.035 | 0.037 | 11 | 0.303 |
| Item 7 | 0.031 | 0.032 | 3 | 0.018 | 0.016 | 0.017 | 4 | 0.212 | 0.014 | 0.015 | 11 | 0.930 |
| Item 9 | 0.023 | 0.024 | 3 | 0.336 | 0.012 | 0.013 | 4 | 0.368 | 0.036 | 0.037 | 11 | 0.292 |
| Item 10 | 0.041 | 0.043 | 3 | 0.023 | 0.005 | 0.005 | 4 | 0.790 | 0.028 | 0.029 | 11 | 0.512 |
| Item 13 | 0.036 | 0.038 | 3 | 0.318 | 0.016 | 0.017 | 4 | 0.203 | 0.028 | 0.030 | 11 | 0.506 |
|  | Perceived Stress (Class Interval) | | | | Education | | | | Perceived Stress-by-Education | | | |
|  | η2 | η_p_2 | *df* | Prob | η2 | η_p_2 | *df* | Prob | η2 | η_p_2 | *df* | Prob |
| Item 4 | 0.106 | 0.107 | 3 | **0.000** | 0.000 | 0.000 | 1 | 0.751 | 0.009 | 0.010 | 3 | 0.328 |
| Item 5 | 0.045 | 0.046 | 3 | 0.009 | 0.001 | 0.001 | 1 | 0.522 | 0.012 | 0.013 | 3 | 0.213 |
| Item 6 | 0.045 | 0.046 | 3 | 0.026 | 0.000 | 0.000 | 1 | 0.879 | 0.001 | 0.002 | 3 | 0.912 |
| Item 7 | 0.031 | 0.031 | 3 | 0.017 | 0.009 | 0.010 | 1 | 0.063 | 0.008 | 0.008 | 3 | 0.396 |
| Item 9 | 0.022 | 0.022 | 3 | 0.370 | 0.005 | 0.005 | 1 | 0.170 | 0.003 | 0.003 | 3 | 0.818 |
| Item 10 | 0.041 | 0.041 | 3 | 0.024 | 0.000 | 0.000 | 1 | 0.837 | 0.014 | 0.014 | 3 | 0.166 |
| Item 13 | 0.020 | 0.021 | 3 | 0.331 | 0.002 | 0.002 | 1 | 0.432 | 0.006 | 0.006 | 3 | 0.532 |
|  | Perceived Stress (Class Interval) | | | | Smoking Status | | | | Perceived Stress-by-Smoking Status | | | |
|  | η2 | η_p_2 | *df* | Prob | η2 | η_p_2 | *df* | Prob | η2 | η_p_2 | *df* | Prob |
| Item 4 | 0.099 | 0.100 | 3 | **0.000** | 0.002 | 0.002 | 2 | 0.672 | 0.005 | 0.005 | 6 | 0.933 |
| Item 5 | 0.032 | 0.033 | 3 | 0.009 | 0.007 | 0.007 | 2 | 0.280 | 0.021 | 0.022 | 6 | 0.258 |
| Item 6 | 0.026 | 0.027 | 3 | 0.025 | 0.003 | 0.003 | 2 | 0.554 | 0.027 | 0.027 | 6 | 0.142 |
| Item 7 | 0.029 | 0.029 | 3 | 0.017 | 0.000 | 0.000 | 2 | 0.953 | 0.017 | 0.018 | 6 | 0.401 |
| Item 9 | 0.009 | 0.009 | 3 | 0.389 | 0.000 | 0.000 | 2 | 0.986 | 0.022 | 0.022 | 6 | 0.253 |
| Item 10 | 0.026 | 0.027 | 3 | 0.025 | 0.008 | 0.008 | 2 | 0.234 | 0.024 | 0.024 | 6 | 0.198 |
| Item 13 | 0.009 | 0.010 | 3 | 0.343 | 0.005 | 0.005 | 2 | 0.386 | 0.011 | 0.011 | 6 | 0.679 |
