# Supplementary material for: Stress beyond coping? A Rasch analysis of the Perceived Stress Scale (PSS-14) in an Aboriginal population
Source: PLoS One. 2019 May 3;14(5):e0216333. doi: 10.1371/journal.pone.0216333 (PMC6499425; doi:10.1371/journal.pone.0216333)
Supplement: S9 Table — i. Every item started with the sentence “How often during the LAST YEAR have you….”. Note. The second column displays the items’ location on the latent trait scale (i.e. the item difficulty). Values of the Fit Residual statistic indicating item misfit (i.e. lower than -2.5 or higher than 2.5), as well as statistically significant χ2 indicating misfit due to item-trait interaction, were highlighted in bold. (DOCX) [file pone.0216333.s009.docx]

**S9 Table.**

| Item^i^ | Location | SE | Fit Residual | *df* | *χ2* | *df* | Prob |
| --- | --- | --- | --- | --- | --- | --- | --- |
| Composite Item 3 | 0.427 | 0.044 | -0.399 | 260.700 | 3.160 | 7.000 | 0.870 |
| Composite Item 4 | -0.044 | 0.044 | -1.161 | 261.430 | 2.670 | 7.000 | 0.914 |
| 9. felt able to control irritations in your life? | -0.338 | 0.064 | 1.482 | 261.430 | 9.332 | 7.000 | 0.230 |
| 13. felt able to control how you spend your time? | -0.046 | 0.061 | 1.501 | 261.430 | 2.472 | 7.000 | 0.929 |
