# Supplementary material for: Stress beyond coping? A Rasch analysis of the Perceived Stress Scale (PSS-14) in an Aboriginal population
Source: PLoS One. 2019 May 3;14(5):e0216333. doi: 10.1371/journal.pone.0216333 (PMC6499425; doi:10.1371/journal.pone.0216333)
Supplement: S10 Table — Note. The table displays the factor loadings of the items responses’ residuals on the first principal component (i.e. the first residual component). (DOCX) [file pone.0216333.s010.docx]

**S10 Table.**

| Item | Composite Item1 | | Composite Item2 | Item8 | Composite Item3 | | Composite Item4 | | Item9 | | Item13 | |
| --- | --- | --- | --- | --- | --- | --- | --- | --- | --- | --- | --- | --- |
| Factor Loading | 0.654 | 0.770 | | 0.399 | | -0.689 | | -0.679 | | -0.665 | | -0.647 |
