# Supplementary material for: Stress beyond coping? A Rasch analysis of the Perceived Stress Scale (PSS-14) in an Aboriginal population
Source: PLoS One. 2019 May 3;14(5):e0216333. doi: 10.1371/journal.pone.0216333 (PMC6499425; doi:10.1371/journal.pone.0216333)
Supplement: S11 Table — Note. The table displays the score distribution of the revised Perceived Distress and Perceived Coping subscales. The items were responded on a five-point Likert scale (0 = Not at all, 1 = Rarely, 2 = Sometimes, 3 = Fairly often, 4 = Very often). (DOCX) [file pone.0216333.s011.docx]

**S11 Table.**

| Perceived Distress subscale | | | Perceived Coping subscale | | |
| --- | --- | --- | --- | --- | --- |
| Total Score | Frequency | Cumulative % | Total Score | Frequency | Cumulative % |
| 0 | 5 | 1.4 | 0 | 7 | 1.9 |
| 2 | 3 | 2.2 | 1 | 6 | 3.5 |
| 3 | 3 | 3.0 | 2 | 3 | 4.4 |
| 4 | 11 | 6.0 | 3 | 11 | 7.4 |
| 5 | 6 | 7.6 | 4 | 10 | 10.1 |
| 6 | 10 | 10.4 | 5 | 16 | 14.4 |
| 7 | 6 | 12.0 | 6 | 28 | 22.1 |
| 8 | 18 | 16.9 | 7 | 28 | 29.7 |
| 9 | 18 | 21.8 | 8 | 35 | 39.2 |
| 10 | 21 | 27.5 | 9 | 30 | 47.4 |
| 11 | 20 | 33.0 | 10 | 27 | 54.8 |
| 12 | 26 | 40.1 | 11 | 34 | 64.0 |
| 13 | 33 | 49.0 | 12 | 33 | 73.0 |
| 14 | 27 | 56.4 | 13 | 26 | 80.1 |
| 15 | 26 | 63.5 | 14 | 18 | 85.0 |
| 16 | 25 | 70.3 | 15 | 14 | 88.8 |
| 17 | 22 | 76.3 | 16 | 8 | 91.0 |
| 18 | 19 | 81.5 | 17 | 6 | 92.6 |
| 19 | 18 | 86.4 | 18 | 10 | 95.4 |
| 20 | 10 | 89.1 | 19 | 3 | 96.2 |
| 21 | 6 | 90.7 | 20 | 3 | 97.0 |
| 22 | 6 | 92.4 | 22 | 3 | 97.8 |
| 23 | 6 | 94.0 | 24 | 1 | 98.1 |
| 24 | 3 | 94.8 |  |  |  |
| 25 | 5 | 96.2 |  |  |  |
| 26 | 1 | 96.5 |  |  |  |
| 27 | 1 | 96.7 |  |  |  |
| Missing | 12 | 100 | Missing | 7 | 100 |
| Total | 367 | 100 | Total | 367 | 100 |
