# Supplementary figures and images for: Stress beyond coping? A Rasch analysis of the Perceived Stress Scale (PSS-14) in an Aboriginal population
Source: PLoS One. 2019 May 3;14(5):e0216333. doi: 10.1371/journal.pone.0216333 (PMC6499425; doi:10.1371/journal.pone.0216333)

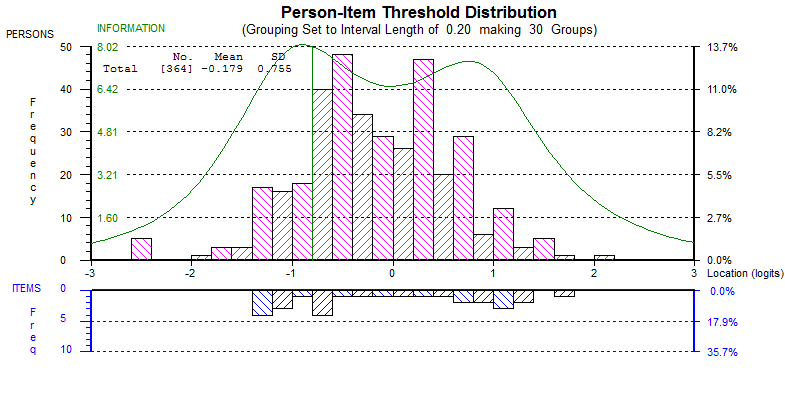

Supplement: S1 Fig — Note. The top bars indicate the distribution of the persons parameters (i.e. “Perceived Distress”) and the bottom bars indicate the distribution of the item thresholds. The Fisher Information function is plotted on the background (i.e. the green line). It should be noticed that the distribution of item thresholds matches the distribution of person parameters throughout the latent trait indicating good targeting of the revised Perceived Distress subscale for this population. (TIFF) [file pone.0216333.s012.tiff]

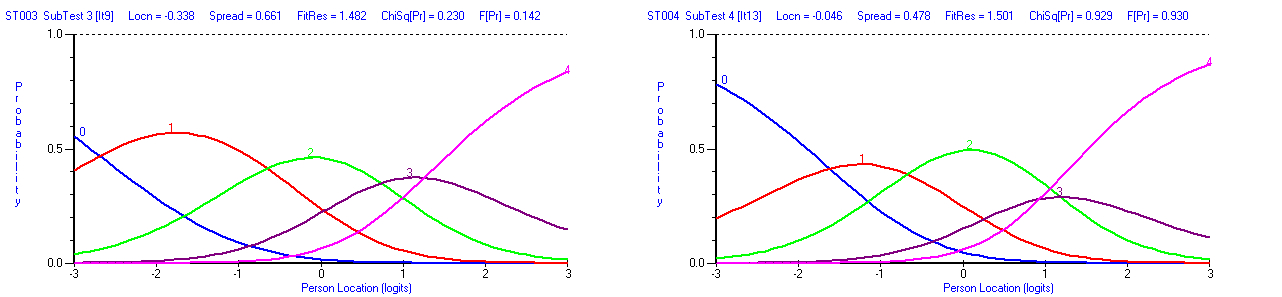

Supplement: S2 Fig — Categories probability curves of Item 9 (left) and Item 13 (right). Note. The graph indicates the probability of endorsing a category according to the latent trait. It can be noticed that for Item 13 category 3 (“Fairly often”) never became the most probable for any range of the latent trait scale. (TIFF) [file pone.0216333.s013.tiff]

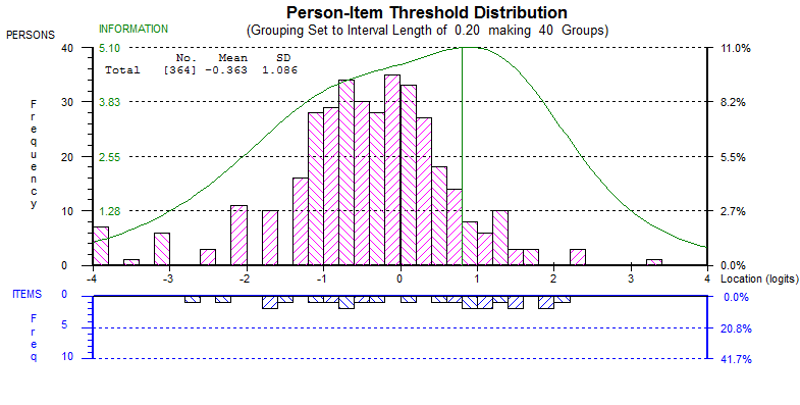

Supplement: S3 Fig — Note. The top bars indicate the distribution of the persons parameters (i.e. “Perceived Coping”) and the bottom bars indicate the distribution of the item thresholds. The Fisher Information function is plotted on the background (i.e. the green line). It can be notice that the distribution of person parameters throughout the latent trait is matched with the distribution of item thresholds indicating good targeting for this population. It should be noticed that the distribution of item thresholds matches the distribution of person parameters throughout the latent trait indicating good targeting of the revised Perceived Coping subscale for this population. (TIFF) [file pone.0216333.s014.tiff]
